# Global disease burden due to antibiotic resistance – state of the evidence

**DOI:** 10.7189/jogh.06.010306

**Published:** 2016-06

**Authors:** Mark Woolhouse, Catriona Waugh, Meghan Rose Perry, Harish Nair

**Affiliations:** 1 Centre for Immunity, Infection and Evolution, University of Edinburgh, Edinburgh, UK; 2 Usher Institute of Population Health Sciences and Informatics, University of Edinburgh, Edinburgh, UK; 3 Regional Infectious Diseases Unit, Western General Hospital, Edinburgh, UK

## BURDEN OF ANTIBIOTIC RESISTANCE

Antimicrobial resistance is widely regarded as one of the major public health concerns of the 21^st^ century [1,2], but there are no good estimates of the net global health burden due to resistance of bacteria to antibiotics. Although numerous studies have provided estimates of the burden of resistance of specific combinations of clinical disease, bacterial agent, antibiotic and health care setting (primarily hospitals in developed countries), metrics vary, coverage is patchy and methodologies are inconsistent. Such data have been used to obtain partial estimates of resistance–related mortality and other outcomes for Europe [3], the USA [4] and the world [5], but because of huge information gaps and the need to extrapolate from small–scale studies these estimates, though helpful, should be regarded as tentative at best.

Multiple metrics are used to quantify the “burden” of infectious diseases, including mortality, morbidity, disability adjusted life years, length of stay in hospital, or cost of care. Here we focus on mortality, although similar considerations apply to other metrics. An essential first step is to provide a clear definition of the burden of antibiotic resistance. We consider the most appropriate definition to be: *the number of deaths attributable to the failure of antibiotic therapy due to antibiotic resistance*. Importantly, this is not equivalent to the total number of deaths among patients with antibiotic resistant infections and may be much less than this for two main reasons: not all patients who may have resistant infections are treated with clinically indicated antibiotics and, for those that are, the measurable difference in outcome for patients with resistant vs susceptible infections may be relatively small.

More formally, this definition of burden can be expressed as a population attributable fraction (PAF, also referred to as the aetiological fraction), ie, the number of deaths that would not occur if antibiotic resistance were eliminated. As set out in the Box, to calculate PAF for mortality due to antibiotic resistance requires data not only on the number of patients with resistant infections and the number that die but also enumeration of the population of interest, which includes patients who survived and/or had susceptible infections. Enumeration of the population of interest in turn requires information on the incidence of the relevant clinical condition, its aetiology, and coverage of the antibiotic therapy of choice. Because such information is rarely available, PAF is rarely used to estimate the global burden of resistance; one recent example considered neonatal sepsis [2], but had to extrapolate key parameters from estimates obtained from a single hospital.

## INCIDENCE AND AETIOLOGY

The main clinical conditions where antibiotic therapy can reduce mortality (**Table 1**) fall into three groups: communicable diseases, endogenous infections and prophylaxis to prevent endogenous infections in high risk patients. The number of patients in these categories defines the population potentially at risk of mortality attributable to antibiotic resistance. Relatively good incidence estimates are available for only some of these categories, notably tuberculosis and health care associated infections [4].

**Table 1:** Common clinical conditions for which antibiotic therapy reduces the risk of mortality

Category of condition	Condition
Communicable diseases	Tuberculosis Sexually transmitted bacterial infections Respiratory bacterial infections (especially of the lower respiratory tract) Diarrhoea caused by bacteria* Healthcare associated bacterial infections
Endogenous infections	Urinary tract infections Skin and soft tissue infections Infective endocarditis Sepsis
Prevention of infection	Burns, wounds Caesarean sections Joint replacements Cancer therapy Organ transplants

*Antibiotics are not necessarily indicated for diarrhoeal cases.

Of the clinical conditions listed in the Table only tuberculosis has a specific aetiology. The remainder are associated with multiple kinds of bacteria and several, such as sexually transmitted infections, diarrhoea and respiratory infections, may also be caused by viral and/or fungal agents.

A restricted set of both gram negative and gram positive bacterial agents, plus *Mycobacterium tuberculosis*, are commonly highlighted in the context of antibiotic resistance (eg, [4]). Some of these are of particular concern in hospital settings, such as *Acinetobacter* spp, *Enterobacteriaceae* spp, *Enterococcus* spp, *Pseudomonas aeruginosa*, *Staphylococcus aureus* and *Streptococcus* spp. Others are associated with communicable diseases typically acquired outside hospitals, such as *Campylobacter* spp, *Neisseria gonorrhoeae*, *Salmonella* *typhi*, non–typhoidal *Salmonella* spp, *Shigella* spp, and *Streptococcus pneumoniae*. Several of these contribute to multiple clinical conditions of interest.

## ANTIBIOTIC USAGE

Global consumption of antibiotics has recently been estimated at more than 70 billion doses per annum [6]. By volume, antibiotic usage in 2010 was dominated by penicillins, cephalosporins, macrolides, fluoroquinolones, trimethoprim and tetracyclines.

These data refer to sales by pharmacies; they do not link antibiotic consumption to the treatment of patients with specific clinical conditions. The WHO last published generic guidelines for the therapeutic use of antibiotics in 2001 [7] but these and more current national and international guidelines tend not to be prescriptive, emphasizing the need to account for local circumstances, not least local patterns of antibiotic resistance. Usage profiles can thus vary considerably between locations. For some countries antibiotic usage data are available at hospital level; again however, these data are not routinely linked to information on the conditions that were being treated [8].

Current antibiotic usage profiles are, of course, influenced by current patterns of antibiotic resistance. Resistance patterns mean that, for example, aminopenicillins alone may not be used to treat serious gram negative bacterial infections, alternative drugs would be used additionally where available. In this scenario, aminopenicillin resistance does not contribute to the population attributable fraction as defined above, although it is arguably an element of the overall burden of antibiotic resistance.

## ANTIBIOTIC RESISTANCE

The most comprehensive data on global levels of antibiotic resistance come from a recent WHO survey [9]. Even so, for most combinations of bacterial species and antibiotic the countries providing the minimum data required (testing of 30 isolates) accounted for less than half the world’s population. A major contribution of this exercise was to highlight significant variations in the kinds of isolates tested and in resistance testing protocols.

Moreover, bacteria–antibiotic combinations were not explicitly linked to clinical condition, so it is unclear when the resistances tested were clinically relevant and when they were not. This, together with the lack of data relating antibiotic usage to clinical condition, makes it difficult to estimate the relevant component of the PAF calculation, the fraction of patients with bacterial infections that are resistant to the antibiotic used to treat them (**Box 1**).

Box 1 **Population attributable fraction (PAF) of mortality due to antibiotic resistance.**

PAF calculations are a standard method of quantifying disease burden associated with a specified risk exposure [2], in this case bacterial infections resistant to the antibiotic used to treat them. The first step is to enumerate the population of interest. For current purposes, this would be the incidence (number per unit time) of patients with one of the clinical conditions of concern (see **Table 1**) and for whom antibiotic therapy is clinically indicated and is provided. The incidence of such patients is denoted *I*.

PAF calculation is routinely expressed in terms of the proportion of population exposed to the risk factor (here, patients with antibiotic–resistant infections) and the risk ratio for mortality standardised to the unexposed group (patients with antibiotic–susceptible infections) [2]. An equivalent, easily understood version is: PAF* = (IF−DR)/(ID−DR)*, where *I* is the overall incidence (number of patients per unit time); *F* is the number of patients with resistant infections that die; *D* is the number of patients that die; *R* is the number of patients with resistant infections. If all deaths are associated with resistance (*F = D*) then *PAF* = 1; if deaths are not disproportionately associated with resistance (corresponding to *F = DR/I*) then PAF = 0. Importantly, PAF = 0 does not equate *F* = 0.

Intuitively, it seems natural to equate *F* with treatment “failures”. However, some care is required because, given PAF<1, it is implicit that some of these patients (estimated as *DR/I*) would have died anyway, even if they had not had a resistant infection (this number reflecting the ‘background’ level of mortality observed in patients who were appropriately treated and had a susceptible infection). Similarly, of patients with susceptible infections who survive, some would have survived anyway, even had they had a resistant infection; that is, not all positive outcomes can be attributed to successful antibiotic therapy.

As detailed in the main text, although there is sometimes information available on *F*, *D* and/or *R*, there is often insufficient information to determine *I*. To do so requires additional data on one or more of the following: i) the total number of patients of interest that survive; ii) the number with susceptible infections; or iii) the number with susceptible infections that survive.

Obtaining a single global estimate of mortality attributable to antibiotic resistance presents the additional challenges of combining and extrapolating estimates of PAF for given combinations of clinical condition, antibiotic, aetiological agent and location, and allowing assessment of future trends.

## TREATMENT FAILURE AND CLINICAL OUTCOME

Two key quantities for estimating the burden of antibiotic resistance are the frequency and clinical impact of failures of antibiotic therapy. Treatment failure is a complex phenomenon that may well be attributable to factors other than antibiotic resistance, including misdiagnosis. Treatment failure can also occur in patients with antibiotic–susceptible infections. Central to the calculation of burden is the distinction between the death of a patient who has an antibiotic resistant infection and the death of a patient that is *attributable* to having an antibiotic resistant infection (see Box).

Data on treatment failures are not routinely recorded. One source of data on mortality is the ICD–10 (International Classification of Disease, version 10) codes used by the WHO [10]. ICD–10 covers many, though not all, of the clinical conditions listed in **Table 1**. However, ICD–10 submissions do not usually include treatment failures associated with antibiotic resistant infections (reference to which is confined to the rarely used “Codes for Special Purposes”). Nor does the Institute for Health Metrics and Evaluation’s Global Burden of Disease cause list have categories linked to antibiotic resistance [11].

## RECOMMENDATIONS

Information currently collected at global or multi–national scales is not sufficient to generate estimates of the disease burden attributable to antibiotic resistance. As a result, current knowledge of the burden of antibiotic resistance is still based largely on the collation of one–off, small–scale, individual studies that vary greatly in setting, scope, sampling frame and methodology, and often requires bold extrapolations to be made from very limited data sets. For estimation of the global burden of antibiotic resistance and, even more, for monitoring changes in burden over time more systematic approaches would be helpful. There are several possibilities.

ICD–10 is due to be replaced by ICD–11 in 2017 [10]. This provides an opportunity to create routinely used categories that record treatment failures, or at least linking treatments with outcomes, the most direct ways to estimate the burden of antibiotic resistance. Specific concerns, such as XDR–TB or carbapenem–resistant *Enterobacteriaceae*, might be prioritised for inclusion.

ICD facilitates passive reporting. An alternative is active reporting by recruiting sentinel sites. For example, 660 hospitals from 67 countries responded to an internet survey on antimicrobial stewardship in 2012 [8]. Monitoring treatment failures due to antibiotic resistance in these hospitals using standardised protocols would generate valuable data. Making selected, high priority antibiotic resistant infections ‘notifiable’ at national level could further improve data capture, extending existing mandatory reporting for specific conditions (for example in the UK for scarlet fever or invasive streptococcal group A disease). Another possibility is a more qualitative approach of recruiting a global panel of individual clinicians who are polled to determine trends in the impact of antibiotic resistance on their patients. Polling has been used successfully in other clinical contexts [12].

As well as estimating the global burden of antibiotic resistance another useful exercise would be to estimate the global burden due to lack of access to suitable antibiotics. For some clinical conditions, this may be a substantially greater burden at the present time [13]. The two issues potentially overlap where there is a lack of knowledge of local resistance profiles (perhaps due to lack of testing facilities) and alternative drugs would have been effective.

**Figure:**
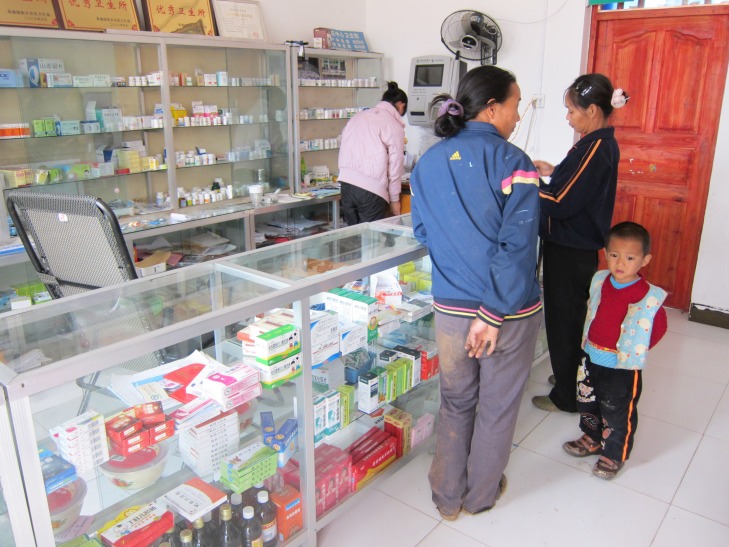
Photo: Courtesy of Kit Yee Chan, personal collection

## CONCLUSIONS

Estimation of the global burden of antibiotic resistance is extremely challenging and arguably not an attainable objective with currently available health data. We stress that this conclusion does not contradict the generally accepted view that antibiotic resistance is a major public health problem of global significance. There is a large number of studies documenting levels of resistance and its clinical impact, and well–founded concerns that both will rise, perhaps dramatically, in the foreseeable future. However, as reviewed here, the valuable insights provided by such studies do not sum to a comprehensive, coherent picture of the global antibiotic resistance burden and how it is changing. Improving this situation will require changes to the ways in which global health statistics are collected; existing approaches are not up to the task. The primary benefit will be more accurate assessment of the global disease burden due to antibiotic resistance and its forward trajectory, helping make the case for investment in combating the problem.
